# Anti-Breast Cancer Properties and In Vivo Safety Profile of a Bis-Carbazole Derivative

**DOI:** 10.3390/pharmaceutics17040415

**Published:** 2025-03-25

**Authors:** Jessica Ceramella, Camillo Rosano, Domenico Iacopetta, Iméne Ben Toumia, Leila Chekir-Ghedira, Mouna Maatouk, Annaluisa Mariconda, Pasquale Longo, Patrick Dallemagne, Christophe Rochais, Maria Stefania Sinicropi

**Affiliations:** 1 Department of Pharmacy, Health and Nutritional Sciences, University of Calabria, Via Pietro Bucci, 87036 Arcavacata di Rende, Italy; jessica.ceramella@unical.it (J.C.); s.sinicropi@unical.it (M.S.S.); 2 U.O. Proteomica e Spettrometria di Massa, IRCCS Ospedale Policlinico San Martino, Largo Rosanna Benzi, 10, 16132 Genova, Italy; camillo.rosano@hsanmartino.it (C.R.); ben.toumia.imene@gmail.com (I.B.T.); 3 Laboratory of Bioactive Natural Substances and Biotechnology, Faculty of Dentistry of Monastir, University of Monastir, Monastir 5000, Tunisia; chekir@yahoo.fr (L.C.-G.); maatoukmouna@yahoo.fr (M.M.); 4 Laboratory of Molecular and Cellular Biology, Faculty of Dental Medicine of Monastir, University of Monastir, Monastir 5000, Tunisia; 5 Department of Basic and Applied Sciences, University of Basilicata, Via dell’Ateneo Lucano, 10, 85100 Potenza, Italy; annaluisa.mariconda@unibas.it; 6 Department of Chemistry and Biology “A. Zambelli”, University of Salerno, Via Giovanni Paolo II, 132, 84084 Fisciano, Italy; plongo@unisa.it; 7 Université Caen Normandie, Normandie University, CERMN UR4258, F-14000 Caen, France; patrick.dallemagne@unicaen.fr (P.D.); christophe.rochais@unicaen.fr (C.R.)

**Keywords:** carbazole derivatives, anticancer, cytoskeleton, apoptosis, toxicity studies

## Abstract

**Background:** Carbazoles represent one of the most important classes of nitrogen-based tricyclic aromatic heterocycles and are present in natural sources and chemically obtained drugs. Recently, several research groups disclosed their large biological and chemical applications in different fields, leading to an increased interest towards this class of molecules. Some of the obtained derivatives have been successfully employed in the clinical treatment of different tumor types, but the onset of heavy side effects impaired their efficacy and discouraged their use. Pursuing the aim of obtaining carbazoles with less negative features, a lot of chemically modified compounds have been produced and evaluated. **Objectives/Methods**: In this paper, we describe the in vitro and in vivo evaluation of a bis-carbazole derivative with strong anticancer properties against two breast cancer cell lines. **Results**: This compound has been found to impact the cell cytoskeleton dynamics, triggering the activation of some key proteins playing a role in the intrinsic and extrinsic apoptotic pathways. Equally important, this derivative has been found to be selective for cancer cells and has shown a safe profile in Balb/c-treated mice. **Conclusions**: Overall, the disclosed outcomes represent an important landmark for encouraging further studies directed toward the potentiation of this lead to be potentially exploited in both preclinical and clinical applications.

## 1. Introduction

**Figure 1:**
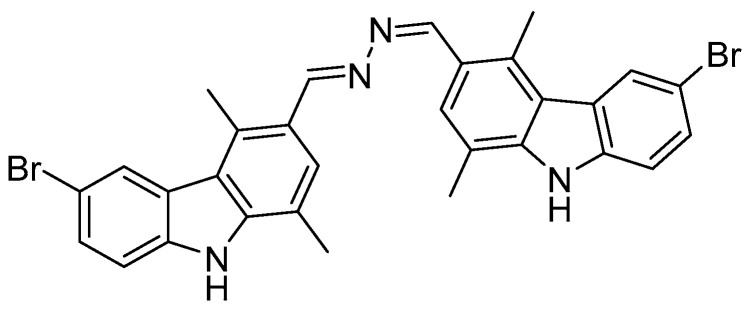
*N*,*N*’-bis-(6-bromo-1,4-dimethyl-9*H*-carbazol-3-ylmethylene)-hydrazine (**1**).

The carbazole scaffold is the key structural motif of many biologically active compounds, including natural and synthetic products [1]. Since the discovery of carbazole-containing molecules in plants from the family of Rutaceae and, as well, from other sources such as bacteria, algae, fungi, and coal tar, several carbazole derivatives were synthesized. Ellipticine is considered the ancestor compound of carbazoles, extracted from *Ochrosia elliptica* leaves and then also chemically synthesized [2]. Carbazoles were biologically investigated for their different activities that include, amongst all, antioxidant, anti-inflammatory, antibacterial, anticancer, antiviral, and antidiabetic ones [3]. Particularly, the anticancer activity of carbazoles is explicated by modulating several targets and relative pathways, as, for instance, DNA and its metabolizing enzymes (topoisomerases and telomerases), kinases, cell cycle proteins, estrogen receptors, cytoskeleton components, apoptotic regulators, and so on [4,5]. With regard to the antitumor activity, three derivatives, *N*-methyl-9-hydroxyellipticinium acetate (Celiptium^®^), alectinib (Alecensa^®^), and midostaurin (Rydapt^®^), obtained the marketing authorization and were clinically used in some countries, revealing their good activity toward different tumor types [6,7,8]. However, the onset of resistance phenomena and heavy side effects represented the major clinical hindrance; thus, the design of new carbazole derivatives that can simultaneously modulate multiple targets and potentially overcome the above-mentioned issues, in vitro and in vivo, is very desirable [9]. Over the last decade, many carbazole derivatives were developed as innovative anticancer candidates, starting from the already individuated leads and modifying the chemical structure or coupling them with other scaffolds [4]. Particularly, the hybrid molecules were demonstrated to be improved chemical entities, able to modulate multiple targets implied in cancer onset and progression [10]. In some cases, the chemical modifications produced compounds with a higher selectivity and safer cytotoxic profile, potentially able to exert fewer unwanted effects and overcome the possible drug resistance phenomena often associated with a single drug administration [9]. An interesting target of anticancer drugs is cell cytoskeleton, mainly composed of microfilaments, microtubules, and intermediate fibers. Microfilaments are formed by actin and are involved in cellular motility, signaling, and interactions with the cellular and extracellular environment; microtubules are made of α and β tubulin subunits and are deputed to maintain cell shape and assure intracellular materials transport; finally, intermediate fibers are tissue-specific and are involved in cell differentiation [11]. In normal conditions, the cytoskeleton plays, thus, important roles in cell metabolism, but when a tumor occurs, the cytoskeleton reorganization plays a main role in cell shape change, migration, and infiltration of tumor cells. In clinical practice, the microtubule-targeting agents (MTAs) are considered amongst the drugs with the highest benefits, and various types of tubulin polymerization modulators have been developed, since the disruption of the microtubule dynamics by MTAs can trigger cancer cell apoptosis [12,13]. The most known examples of MTAs are Vinblastine, which induces microtubule depolymerization, and Paclitaxel, a microtubule stabilizer that increases polymerization reaction [14]. MTAs have several applications, particularly for facing cancer development and metastasization. However, two main problems emerged from cancer therapy with MTAs, namely the high systemic toxicity and the onset of resistance phenomena [15]. The latter represent a major hindrance for the clinical success of MTAs and often rise from the overexpression of the P-glycoprotein (P-gp), able to actively transport xenobiotics out of cells, tubulin mutations, and/or the expression of tubulin isotypes, which are less sensible to MTAs [16]. Resistance phenomena may be overthrown using compounds bearing chemical scaffolds or substituents different from the classical taxanes, which bind different tubulin sites or are not recognized by P-gP [15]. In this context, carbazole derivatives were proved to target cytoskeleton components, diminishing the viability and the migratory ability of metastatic cancer cells [5,17,18]. Following the results obtained from our research group [19], we aimed at investigating the anticancer activity of a bis-carbazole derivative, *N*,*N*’-bis-(6-bromo-1,4-dimethyl-9*H*-carbazol-3-ylmethylene)-hydrazine, (**1**, Figure 1), and its ability to target cytoskeleton components.

Particularly, this compound exerted a high activity on two breast cancer cell lines, the triple-negative MDA MB-231 and ER-positive MCF-7, and selectivity with respect to their normal counterpart, the human mammary epithelial cells MCF-10A. This compound was found to be very active toward the MDA-MB-231 cells and able to trigger the activation of caspase-8 and -9, which are relative to the two known apoptotic mechanisms [20], and modulate the activation of the pro-apoptotic protein Bid [21]. The **1**-induced cancer cell death was caused, at least, by the interference with cell cytoskeleton components, as suggested by in silico and in vitro studies. Indeed, compound **1** treatment was able to block the cytoskeleton dynamics by stabilizing the microtubule formation and, consequently, perturbing the actin network as well. These features strongly denote the high potential of **1** as an anticancer drug candidate. An important step in drug development is represented by early in vivo preclinical toxicity studies that may shed light on potential unwanted effects and estimate whether a given compound could be safe for human use or not [22]. For this purpose, in vivo studies were conducted using Balb/c mice administered intraperitoneally for one week with **1**. The obtained outcomes did not evidence significant differences in organ weight, hepatotoxicity, and nephrotoxicity markers between the compound **1**-treated group and the negative control group. Moreover, hematological analyses suggested, as well, a lack of any toxicity at the adopted experimental conditions. Summing up, these results pave the way for further development and in-depth studies regarding other possible mechanisms of action and in vivo efficacy in induced tumors and strongly suggest compound **1** as a powerful and safe lead for treating metastatic cancer.

## 2. Materials and Methods

### 2.1. Cell Cultures

The three cell lines adopted in this work (MCF-7, MDA-MB-231 and MCF-10A) were obtained from the American Type Culture Collection (ATCC, Manassas, VA, USA). MCF-7 human breast cancer cells were cultured in Dulbecco’s modified Eagle’s medium/nutrient mixture Ham F-12 (DMEM/F12), added with 10% fetal bovine serum (FBS) and 100 U mL^−1^ penicillin/streptomycin. MDA-MB-231 human breast cancer cells were maintained in DMEM/F12 added with 5% FBS, 1% L-glutamine and 100 U mL^−1^ penicillin/streptomycin. MCF-10A human mammary epithelial cells were cultured in DMEM/F12 medium added with 5% horse serum (HS, Thermo Fisher Scientific, Milan, Italy), 100 U mL^−1^ penicillin/streptomycin, 0.5 mg mL^−1^ hydrocortisone, 20 ng mL^−1^ human epidermal growth factor (hEGF), 10 mg mL^−1^ insulin, and 0.1 mg mL^−1^ cholera enterotoxin (Sigma–Aldrich, Milan, Italy). Cells were cultured at 37 °C in a humidified atmosphere (95% air and 5% CO_2_) and periodically screened for checking contamination [23].

### 2.2. Cell Viability

The 3-(4,5-dimethylthiazol-2-y1)-2,5-diphenyltetrazolium Bromide (MTT, Sigma–Aldrich, Milan, Italy) assay was used for determining cell viability [24]. Cells were seeded on forty-eight well plates and cultured in a complete medium. Next, cells were serum-deprived (24 h). Then, cells were exposed to the tested compounds in phenol-red-free medium added with 1% of serum for 72 h. After medium removal, MTT solution was added to each well (0.5 mg/mL) and incubated at 37 °C (2 h). Cells were lysed (DMSO), and the optical density was measured (570 nm) using a microplate reader. Mean absorbance was reported as a percentage versus control, and IC_50_ values were calculated using GraphPad Prism 8 software (version 8.4.3., GraphPad Inc., San Diego, CA, USA). The selectivity index was obtained by the ratio of IC_50_ values calculated for normal and cancer cells. Data represent three independent experiments; standard deviations (SD) were reported.

### 2.3. TUNEL Assay

The TUNEL assay was used for apoptosis detection, according to the manufacturer’s instructions (CF^TM^488A TUNEL Assay Apoptosis Detection Kit, Biotium, Hayward, CA, USA), with some modifications (ref). Shortly, cells were grown on glass coverslips, then treated with **1** at its IC_50_ value. After that, they were PBS washed thrice, then methanol-fixed (−20 °C, 15 min). Cells were fixed and incubated with the enzyme and, next, counterstained with DAPI (0.2 mg mL^−1^) (10 min, room temperature). Cells were washed three times with cold PBS; the mounting solution was added and observed and imaged using a fluorescence microscope (Leica DM6000, Leica Microsystems Srl, Milan, I-20090, Italy; 20× magnification) with excitation/emission wavelength maxima of 490 nm/515 nm (CF^TM^488A) or 350 nm/460 nm (DAPI) [25]. Images represent two independent experiments, and representative fields were shown.

### 2.4. Caspases Assay

Caspases-3/7, -8, and -9 activities were determined using the Caspase-Glo^®^ 3/7, 8, and 9 Assay Systems (Promega Corporation, Madison, WI, USA) as previously described [26].

### 2.5. Immunofluorescence Studies and Mitochondria Staining

Cells grown on glass coverslips in full medium were serum-deprived for 24 h and exposed to 1 for 24 h at its IC_50_ value concentration. Cells were PBS-washed and fixed (cold methanol, 15 min, −20 °C), then primary antibody in blocking solution was added (4 °C/overnight), as described [27]. The mouse anti-Bid (sc-373939) and anti-β-Actin (69879) and the rabbit anti-β Tubulin (9104) were obtained from Santa Cruz Biotechnology and employed at a 1:100 dilution. Coverslips were washed thrice with PBS, then the secondary antibody Alexa Fluor^®^ 568 conjugate goat-anti-mouse and Alexa Fluor^®^ 488 conjugate goat-anti-rabbit (1:500, Thermo Fisher Scientific, Waltham, MA, USA) was added. Nuclei were DAPI (Sigma Aldrich, Mila, Italy) stained (10 min, 0.2 μg/mL) and PBS washed thrice. For mitochondrial staining, cells were pre-incubated with prewarmed (37 °C) MitoTracker^®^ Deep Red FM probe solution (MitoTracker^®^ Mitochondrion-Selective Probes, Invitrogen European Headquarters, Paisley PA4 9RF, UK) for 20 min (fluorescence excitation = 644, fluorescence emission = 665). A fluorescence microscope (Leica DM 6000) and LAS-X software (Leica Application Suite X, 3.5.7.23225) were used to acquire and process all images.

### 2.6. In Vitro Tubulin Polymerization Assay

The tubulin polymerization inhibition was detected using the in vitro Tubulin Polymerization Assay Kit (EMD Millipore Corporation, Burlington, MA, USA), following the procedure previously published [28]. Briefly, polymerization reactions were performed in 70 µL final volumes (60 µL of the 60 µM tubulin in 1xPB-GTP and 10 µL of the test substance dissolved in 1xPB-GTP). Paclitaxel and Vinblastine (both at 10 µM) and **1** (1 µM) were dissolved in DMSO. The turbidity variation was measured every 30 s at 350 nm for 90 min. Turbidity (absorbance) readings were used to determine the extent of polymerization [% inhibition = (1 − A_350_ sample/A_350_ control) × 100].

### 2.7. Molecular Docking

Molecular docking simulations were performed to study the interaction between the compound **1** and tubulin, using a protocol described in several previous publications [28,29]. The three-dimensional structure of tubulin was retrieved from the Protein Data Bank [PDB ID: 7Z6S] [30] and prepared using AutoDock Tools [31] by removing all water molecules, adding polar hydrogens, and assigning Gasteiger charges. The compound **1** was constructed and optimized using MarvinSketch [ChemAxon Ltd., Budapest, Hungary], ensuring proper geometry and flexibility.

Docking simulations were carried out with AutoDock 4.2 [32], employing the Lamarckian genetic algorithm. A grid box was defined to include the binding regions of tubulin, with dimensions large enough to allow an unbiased search for potential interactions. The grid spacing was set to 0.375 Å, and 100 independent runs were performed for each simulation, with a population size of 150 and up to 2.5 million energy evaluations per run.

The docking results were clustered based on a root mean square deviation (RMSD) cutoff of 2.0 Å, and binding energies were calculated for ranking the poses. Molecular interactions and docking poses were visualized and analyzed using Chimera [33], which was also used to generate all publication-quality

### 2.8. In Vivo Studies

Ethical Approval: All experimental procedures were carried out in accordance with the National Institutes of Health guidelines for the care and use of laboratory animals (NIH, 2011). Prior to the commencement of the study, ethical approval was obtained from the Animal Ethics Committee in Tunisia, ensuring that all experimental protocols adhered to the highest standards of animal welfare.

Animals: The study utilized male Balb/c mice aged 6–8 weeks, with weights ranging from 22 to 26 g. These mice were sourced from a reputable supplier and maintained in a certified pathogen-free facility. They were housed in polycarbonate cages under controlled environmental conditions (temperature: 20–22 °C; humidity: 40–60%) with a 12 h light/dark cycle. Mice had ad libitum access to filtered water and a standard rodent chow diet, ensuring their nutritional needs were met throughout the study.

Preparation of Test Item: The investigational solution that contains the compound **1** was prepared before administration to ensure its stability and efficacy. The preparation involved dissolving compound **1** in a solvent mixture comprising 5% ethanol and Cremophor, diluted in 95% phosphate-buffered saline (PBS). The choice of this solvent system was based on previous studies demonstrating its compatibility with similar compounds [34]. All solutions were prepared in a sterile environment to minimize the risk of contamination.

Experimental Design: Mice were randomly assigned to one of two experimental groups to eliminate bias in treatment effects. Group 1 (negative control) consisted of six mice that received intraperitoneal injections of the vehicle solvent (5% ethanol–Cremophor in 95% PBS). Group 2 (compound **1** treatment group) comprised seven mice receiving compound **1** at a dosage of 20 mg/kg body weight, administered via intraperitoneal injection. The dosage was selected based on preliminary dose–response studies ensuring safety and efficacy.

Treatment Protocol: The treatment regimen involved administering injections every two days over a one-week period, culminating in a total of three injections per mouse. This schedule was designed to maintain consistent therapeutic levels of compound **1** in the bloodstream, providing sufficient time for both pharmacological effects and assessment of any potential adverse reactions.

Assessment Parameters: Behavioral observations were meticulously recorded on a daily basis throughout the treatment period. Trained personnel monitored the mice for signs of toxicity or any deviations from normal behavior, including changes in activity levels, grooming patterns, and feeding behavior [35]. Body weight was measured at baseline prior to treatment, immediately before each injection, and at the conclusion of the treatment period to assess any potential effects of compound **1** on growth and overall health.

After the treatment phase, mice were humanely euthanized using approved methods to minimize distress. Major organs, including the spleen, liver, lungs, brain, heart, and kidneys, were carefully excised, weighed, and subjected to morphological examination to identify any structural abnormalities or pathological changes. The organ coefficients were calculated using the following formula:

Organ Coefficient (mg/10 g) = 10 × body weight/weight of the mouse.

This calculation allowed for the assessment of organ growth relative to overall body mass.

Blood samples were collected post-euthanasia using heparinized tubes to prevent coagulation. Following collection, samples were centrifuged at 1500 rpm for 7 min to separate the plasma, which was then used for biochemical analysis. A clinical chemistry analyzer measured various parameters, including transaminases (AST, ALT), creatinine, uric acid, and key electrolytes (sodium, potassium, chloride, calcium, and phosphorus), alongside alkaline phosphatase levels. Hematological evaluations were performed, including red blood cell (RBC) counts, hemoglobin (HB) levels, hematocrit (HCT), mean corpuscular hemoglobin (MCH), and white blood cell (WBC) counts [36,37].

### 2.9. Hydrolytic Stability Test

^1^H NMR spectra were recorded at 298 K on a Bruker Avance 400 spectrometer operating at 400 MHz (^1^H) and referred to the residual proton deuterated solvent peaks (^1^H: (CD_3_)_2_SO, δ 2.50).

### 2.10. Statistical Analysis

Statistical analysis was conducted using appropriate methods to assess the significance of findings. Biological in vitro data were analyzed for statistical significance using one-way ANOVA followed by Dunnett’s test, performed by GraphPad Prism 8. Standard deviations (SD) are shown. For in vivo studies, comparisons between treatment groups were performed using t-tests, with a significance level set at *p* < 0.05. All data are presented as mean ± standard deviation (SD), allowing for a clear understanding of variability and ensuring the reliability of the results. Graphical representations of data were employed where appropriate to enhance interpretability.

## 3. Results and Discussion

### 3.1. Anticancer Activity and DNA Damage

The cytotoxic potential of the bis-carbazole compound **1**, synthesized as previously published [19], was investigated adopting two breast cancer cell lines, the ER-positive MCF-7 and triple-negative MDA-MB-231, and their normal counterpart, *viz.*, the human mammary epithelial cells MCF-10A. MTT test was employed, and the obtained IC_50_ values, expressed as μM, were resumed in Table 1, together with the calculated selectivity index (SI). Ellipticine, the most known carbazole scaffold-containing molecule, was used as a reference for viability assays. As can be observed from Table 1, the compound **1** IC_50_ values fall in the low micromolar range, indicating a high activity against both the cancer cells used in these assays, with a little difference. Indeed, compound **1** possessed the best activity on the MDA-MB-231 cells, with an IC_50_ value of 0.3 ± 0.2 μM, which is very close to that calculated for MCF-7 cells since, in this case, the IC_50_ value was 0.6 ± 0.3 μM. Furthermore, compound **1** lacked any cytotoxic activity against the normal cell lines used in these experiments (see Table 1, IC_50_ > 100 μM). The calculated SI values indicated an extraordinarily higher selectivity toward both the breast cancer cells with respect to the normal ones, as visible in Table 1. Ellipticine greatly impacted the MCF-7 and MDA-MB-231 breast cancer cells viability, with IC_50_ values of 1.3 ± 0.2 and 1.9 ± 0.1 μM, respectively, but exerted a very similar effect on the normal MCF-10A cells, where the calculated IC_50_ value was 1.2 ± 0.3 μM (Table 1). Effectively, Ellipticine showed a lack of selectivity, as indicated by its SI values of 0.9 and 0.8 for MCF-7 and MDA-MB-231 cells, respectively. It should be highlighted that **1** exhibited a higher anticancer activity toward both the breast cancer cells compared to Ellipticine, being two- and six-fold more potent, together with a complete lack of cytotoxicity against the normal MCF-10A cells, as indicated by the calculated SI values. Furthermore, Vinblastine and Paclitaxel were also included. As visible in Table 1, both the compounds exhibited lower IC_50_ values but also a lower selectivity with respect to compound **1**. Summing up, the exciting anticancer properties make this compound a very interesting lead, of which several proper structural modifications could be made, aiming at obtaining a series of new derivatives to be used for further investigations.

**Figure 2:**
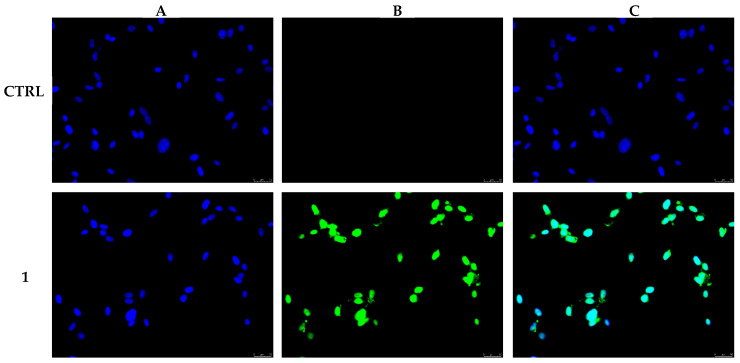
MDA-MB-231 cells were exposed to 0.3 µM of compound **1** or vehicle (CTRL) for 24 h. After processing, cells were observed, and images were taken at 20× magnification by means of an inverted fluorescence microscope. The green fluorescence, visible only for compound **1**-treated cells, indicates nuclear DNA damage. Panels (**A**): DAPI (CTRL and **1**) excitation/emission wavelength 350 nm/460 nm. Panels (**B**): CF™488A (CTRL and **1**) excitation/emission wavelength 490 nm/515 nm. Panels (**C**): panels (**A**) and (**B**) overlay. Images are representative of three separate experiments.

It is widely reported that carbazole derivatives may target several pathways by which they exert their anticancer activity and push cancer cells to death by apoptosis. Amongst the main cellular targets involved in cancer onset and progression, carbazole derivatives were proven to modulate topoisomerases, telomerase, tubulin, caspases, and kinases [2,38]. Much of the literature data reported that Ellipticine and many derivatives induce nuclear DNA damage through different pathways [2,39]; thus, in order to investigate whether **1** could explicate the observed anticancer activity through the nuclear DNA damage and, successively, cell death by apoptosis, a TUNEL assay was performed using MDA-MB-231 cells. Shortly, cells were exposed to **1** at a concentration equal to 0.3 µM or vehicle (DMSO) for 24 h, then subjected to the TUNEL assay, as detailed in the Section 2. As visible in Figure 2, cells treated with **1** were found TUNEL-positive; indeed, the clear green fluorescence (Figure 2, panel B) and the blue fluorescence associated with cell nuclei (Figure 2, panel A) perfectly overlap, indicating massive genomic DNA damage (Figure 2, panel C). Conversely, in MDA-MB-231 cells treated with the only vehicle (DMSO, CTRL), the green fluorescence was not observed (Figure 2, panels B, CTRL), meaning that the nuclear DNA was undamaged (Figure 2, panels A, CTRL).

### 3.2. Compound **1** Treatment Induces MDA-MB-231 Breast Cancer Cells Apoptosis

**Figure 3:**
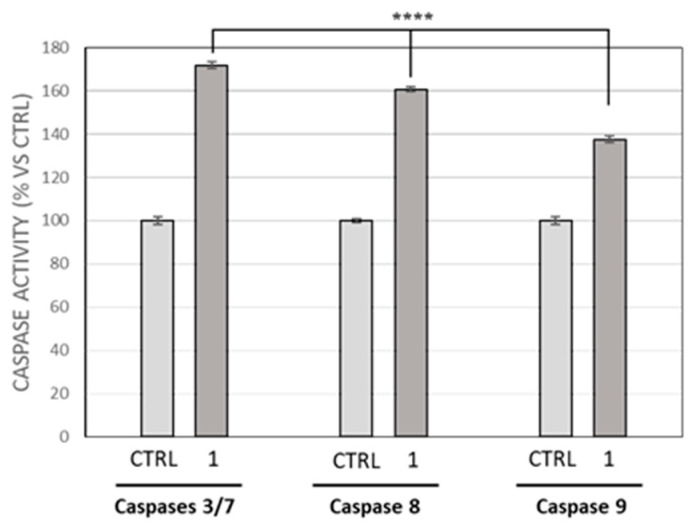
Determination of caspase-3/7, -8, and -9 activity levels following the treatment of MDA-MB-231 cells with compound **1** (0.3 µM) for 24 h, reported as a percentage versus the DMSO-treated cells, used as a control (CTRL). Data represent the means ± SD of three different experiments. **** *p* < 0.0001, treated versus CTRL.

One of the most determinant components of an effective anticancer drug is represented by the induction of apoptosis, which is an important event also connected with the onset of drug resistance phenomena [40]. Considering the previous data about the nuclear DNA damage and in order to determine whether MDA-MB-231 cells were undergoing apoptosis after compound **1** treatment, the activity of the initiator caspase-8 and -9 and the executioner caspase-3 and -7 was measured. The obtained outcomes are reported in Figure 3, where it is possible to see that the activity of all the caspases was found to have risen in MDA-MB-231 cells treated with 0.3 µM of compound **1** for 24 h, compared to the DMSO-treated cells (vehicle). Particularly, an increase of about 70% was found for the activity of caspase-3 and -7, which are usually cleaved and activated by the initiator caspase-8; the latter showed an activity increase of about 60%, whereas a 40% was recorded for the caspase-9.

**Figure 4:**
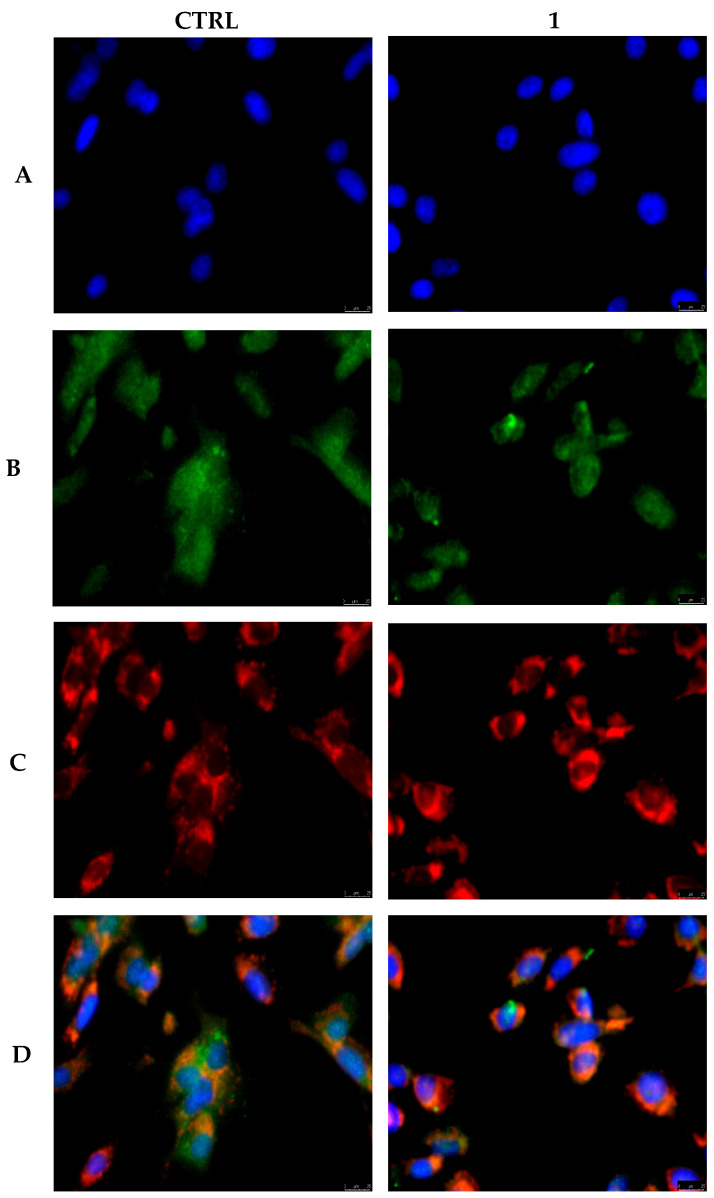
MDA-MB-231 cells were treated with 0.3 µM of **1** or vehicle (CTRL) for 24 h, then processed as described in the experimental section and observed at 40× magnification using an inverted fluorescence microscope. Compound **1** treatment induced Bid (green fluorescence, panel (**B**), **1**) translocation into mitochondria (red fluorescence, panel (**C**), **1**), as noticeable in the merge channel (panel (**D**), **1**). Nucleus staining is also shown (panels (**A**)). Panels (**A**): DAPI, excitation/emission wavelength 350 nm/460 nm. Panels (B): Alexa Fluor^®^ 488, excitation/emission wavelength 490 nm/515 nm. Panel (**C**): MitoTracker Deep Red FM probe, excitation/emission wavelength 644/665 nm. Panels (**D**): panels (**A**) and (**B**) overlay. Representative fields of three different experiments are shown.

It has been widely investigated that the anticancer drugs-induced apoptotic cell death may follow two classic signaling pathways, extrinsic and intrinsic [41,42]. The first is a caspase-dependent pathway, usually mediated by caspase-8 and triggered by extracellular stimulations induced by the recognition of specific membrane receptors and then propagated. The intrinsic pathway, which involves mitochondria, can be caspase-dependent or -independent and can be initiated by different intracellular stimulations through caspase-9 activation [40]. However, both pathways lead to the cleavage of the downstream executioner caspase-3 and -7, ultimately inducing apoptosis [43]. Considering that MDA-MB-231 cancer cells exposed to **1** showed nuclear DNA damage and the rise in both caspase-8 and -9 activity, it should be postulated that both the apoptotic pathways are taking place or that this compound could stimulate the membrane death receptors. The latter event usually leads to pro-caspase-8 activation that, in turn, directly activates caspase-3 or induces the mitochondrial pathway through the release of Cytochrome c from mitochondria and procaspase-9 activation [44]. In recent studies of apoptotic models, the activation of the mitochondrial arm of the above-mentioned pathway follows the activation of a death receptor. The bridging element between the two arms of the apoptosis is the pro-apoptotic protein Bid, a member of Bcl-2 protein family, which cleavage is mediated by caspase-8 [21]. Our observations suggest that, following compound **1** treatment, the death receptors activate caspase-8 that cleaves Bid, whose truncated form is able to induce mitochondrial outer membrane permeabilization, Cytochrome c release, apoptosome formation, and procaspase-9 cleavage, leading to the activation of executioner caspase-3/7 and death by apoptosis. In order to obtain further information about the mechanism induced by **1**, the status of Bid was investigated by means of immunofluorescence assays in MDA-MB-231 cells exposed to the compound **1** for 24 h at 0.3 µM. As visible in Figure 4, in vehicle-treated cells, the green fluorescence associated with Bid is widely diffused within the cell cytoplasm (panel B, CTRL), and the mitochondrial network (red fluorescence, panel C, CTRL) looks intact and normally distributed. The overlay panel (D, CTRL) confirms that Bid is not localized within the mitochondria in cells treated with the vehicle only. Contrarily, the exposure of cells to **1** produced a net translocation of Bid into the mitochondrial compartment, particularly visible in panel C (**1**). Indeed, the green fluorescence associated with Bid (panel B, **1**) diminished and seemed mostly confined to the mitochondria (panel C, **1**). Additionally, the latter appeared unevenly distributed and with a more compacted structure, suggesting a loss of the normal function, contrary to what happened in the absence of **1** exposure (panel C, CTRL). These observations well correlate with the detected raise in caspase-8 activity that, cleaving Bid, allowed the formation of an apoptosome and activation of caspase-9, which concurs with the apoptotic cascade producing cancer cell death.

### 3.3. Docking Studies

**Figure 5:**
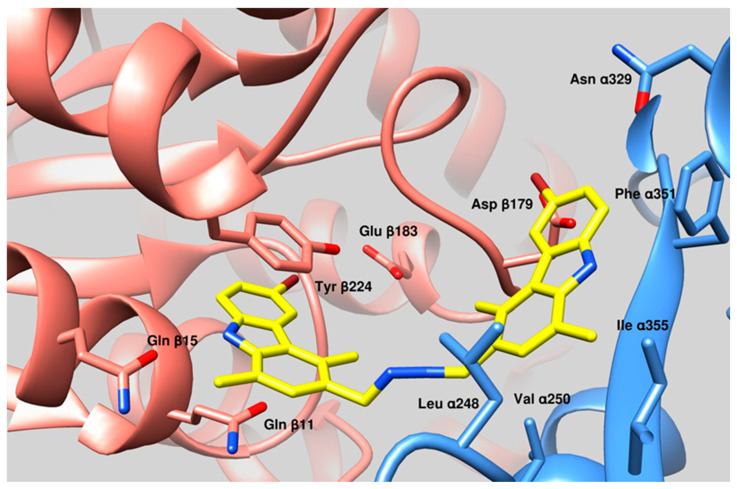
Docking simulations suggested that **1** binds a region proximal to the Paclitaxel-binding site of tubulin. The most important amino acid residues involved in the interactions are evidenced. Cyan ribbons: Tubulin, α-subunit, Salmon ribbon Tubulin β-subunit. Yellow sticks, **1** binding mode. Oxygen atoms belonging to evidenced aminoacids are colored in red, while nitrogen in blue. Bromide moieties are evidenced in amaranth.

The docking simulations revealed that **1** binds the tubulin interface between the β- and α-subunits, a region proximal to the Paclitaxel-binding site (Figure 5). At this interface, **1** exhibited a calculated binding energy of −10.49 kcal/mol and an estimated inhibition constant (Ki) of 20.35 nM. This interaction appears to stabilize the polymerization of tubulin in a manner comparable to Paclitaxel, indicating a similar mechanism of action. Compound **1** forms several key interactions within the β/α-tubulin interface. In the β-subunit, hydrogen bonds are established with Gln β11 and Gln β15, while halogen bonds involve Lys β176 and Glu β183. Additionally, a π–π stacking interaction occurs between the carbazole group of **1** and Tyr β224. In the α-subunit, hydrophobic interactions with Leu α248, Val α250, Val α353, and Ile α355 further contribute to the stabilization of the compound within the binding site. These results underscore the strong binding affinity and the robust interaction network of **1** at the critical β/α-tubulin interface, highlighting its potential as a microtubule-stabilizing agent.

### 3.4. Compound **1** Interferes with MDA-MB-231 Cells Cytoskeleton Dynamics

**Figure 6:**
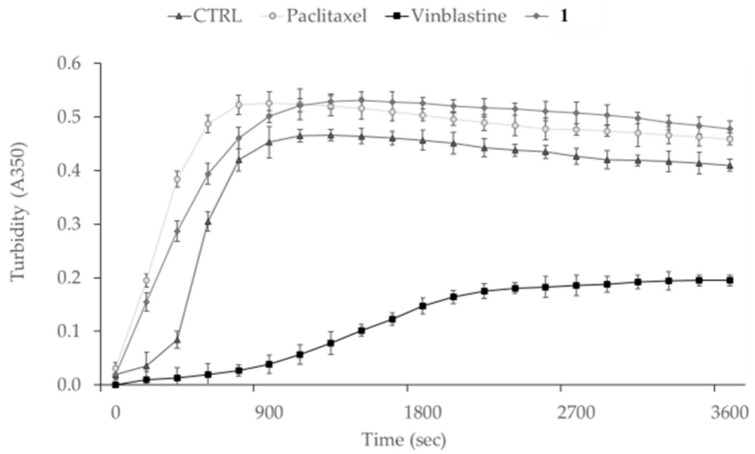
In vitro tubulin polymerization assay. The assembly of tubulin into microtubules was followed by measuring the turbidity (absorbance, A) at 350 nm for 3600 s at 37 °C. The polymerization curves, in the presence of **1** (1 μM) or not (CTRL, DMSO), are shown. Moreover, the graphic shows the curves obtained using two reference molecules, Vinblastine and Paclitaxel, (both at 10 μM concentration), used as tubulin-destabilizing and tubulin-stabilizing agents, respectively.

In recent years, different carbazole scaffold-containing molecules were synthesized and screened for their biological properties and applications, for instance, as anticancer, antioxidant, anti-inflammatory, antimicrobial, and so on [5,10,45,46]. Particularly regarding the anticancer properties, carbazole derivatives were found to impact the cell cytoskeleton dynamics, targeting the microtubule and actin networks and leading to cell cycle arrest and cell death by apoptosis [5,47]. It is noteworthy that the microtubule network plays a main role in cell division but is also involved, to name but a few, in the maintenance of cell morphology, motility, and intracellular trafficking [14]. However, the cytoskeleton modifications can lead to the achievement of tumor cell immortality, which can also acquire migratory properties and higher plasticity, leading to uncontrolled proliferation and metastases formation [48]. Therefore, it is vital to block these events with specific and selective drugs. To date, the most known drugs used in cancer treatment that interfere with the microtubule dynamics are Vinca alkaloids and taxanes [49]. The first class includes Vinblastine, which is a microtubule destabilizing agent because it causes their depolymerization, whereas taxanes, e.g., Paclitaxel, belong to the second class and induce microtubule stabilization, increasing the tubulin polymerization reaction [14]. Preliminary docking studies suggested that **1** binds to tubulin and increases the polymerization reaction, producing the same final effect as Paclitaxel. Thus, aiming at investigating whether **1** could target tubulin, we performed in vitro tubulin polymerization and immunofluorescence assays on MDA-MB-231 cells. In vitro tubulin-polymerization inhibition assay is a well-exploited test for investigating the ability of a given compound to bind tubulin and inhibit or increase the polymerization reaction, measuring the variation of turbidity at 350 nm. In the present test, we adopted Vinblastine and Paclitaxel, as reference molecules for the destabilization or stabilization processes, respectively, at a concentration of 10 µM and the compound under study (**1**) at a concentration of 1 µM. Moreover, a control reaction, using the vehicle (DMSO), was also included. The adopted concentration was chosen after preliminary experiments that were performed in order to individuate the lower concentration of **1** able to produce the maximum effect on the tubulin polymerization reaction. Particularly, we previously employed 10, 5, 1, 0.5, and 0.1 µM of **1**, observing no differences between 10 and 1 µM and a dose-dependent decrease of the effect at doses lower than 1 µM. The obtained outcomes, shown in Figure 6, indicate a regular polymerization curve for the control reaction (DMSO), where the time-dependent increase in turbidity suggests that the tubulin heterodimers self-assembled, forming the microtubules. At the end of the reaction, the optical density value, measured at 350 nm (OD_350_), was about 0.41. The presence of the microtubule-stabilizing agent Paclitaxel in the reaction mixture produced a similar polymerization curve, but with a higher rate in the first 15 min and reaching a final OD_350_ value of about 0.46, indicating a faster assembly and increased amount of tubulin heterodimers. On the contrary, in the presence of Vinblastine, the reaction rate was much lower and reached the steady state later, after about 35–40 min, with a final turbidity value of about 0.2, which equals half of that observed in the control reaction (vehicle). As expected, the tubulin-polymerization reaction was strongly inhibited by Vinblastine. Finally, the exposure of tubulin to **1** did not hamper the tubulin-polymerization reaction but, on the contrary, the reaction curve was closer to that determined by Paclitaxel, with a faster initial rate and reaching the plateau in a similar manner. Moreover, the final OD_350_ value was 0.48, which is higher than that of the control reaction and similar to that of Paclitaxel. These data strongly indicate that **1** acts as a microtubule stabilizer, similarly to Paclitaxel, but with a ten-fold higher potency, considering the adopted concentrations.

**Figure 7:**
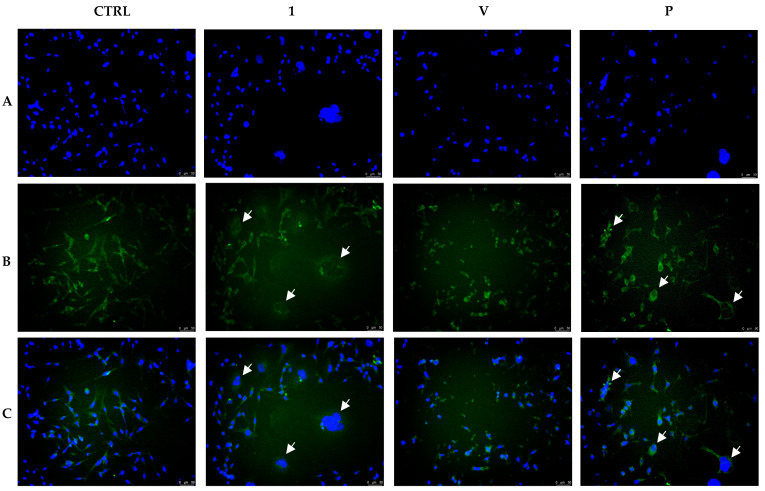
Immunostaining studies on MDA-MB-231 cells treated with compound **1** (0.3 µM), Vinblastine, Paclitaxel (both at 1 µM), or vehicle (CTRL, DMSO) for 24 h. In the control experiment (CTRL), cells exhibited a regular arrangement of cytoskeleton. Vinblastine and Paclitaxel, instead, dramatically impacted the tubulin network. Vinblastine produced tubulin crystal formation (panel (**B**), V), whereas Paclitaxel induced tubulin bundles and thickened fibers (panel (**B**), P). Compound **1** exposure produced a morphology similar to Paclitaxel treatment (white arrows). Panels (**A**): DAPI, excitation/emission wavelength 350 nm/460 nm. Panels (**B**): β-tubulin (Alexa Fluor^®^ 488) excitation/emission wavelength 490 nm/515 nm. Panels (**C**): overlay. Images were taken at 20×, and representative fields were shown.

Aiming at obtaining additional evidence about the ability of **1** in regulating the cell microtubule network, we performed immunofluorescence studies on MDA-MB-231 cells. For this purpose, cells were exposed to 0.3 µM of compound **1**, vehicle (DMSO), Vinblastine, or Paclitaxel, each at 1 µM, for 24 h, then processed as detailed in the experimental section. The results are shown in Figure 7, where it is possible to see that, in the vehicle-treated cells (panel B, CTRL), microtubules appeared filiform and evenly distributed into cell cytoplasm, suggesting a regular organization of the microtubule network. Under Vinblastine (V) treatment, the microtubules appeared thicker and tended to accumulate in disorganized dotted structures (i.e., crystals) close to the cell nuclei (Figure 7, panel B, V). Conversely, the exposure of MDA-MB-231 cells to Paclitaxel induced a massive formation of tubulin bundles and bulker fibers (Figure 7, panel B, P) due to the microtubules non-reversible polymerization. Furthermore, it is possible to see the presence of multinucleated cells with condensed nuclei (indicated by white arrows) because of the mitotic arrest caused by the induced stabilization of microtubules, which is another sign that the apoptotic process is occurring. Finally, **1** treatment induced a net disorganization of the microtubule network, as well, accompanied by the appearance of multilobed nuclei and multiple micronuclei, as visible in Figure 7, panel B (white arrows). Thus, it can be deduced that **1** treatment induced morphological changes similar to those triggered by Paclitaxel in MDA-MB-231 cells. These observations strongly indicated that **1** is a tubulin-polymerization stabilizing agent and are in agreement with the previous results obtained with the polymerization assay.

**Figure 8:**
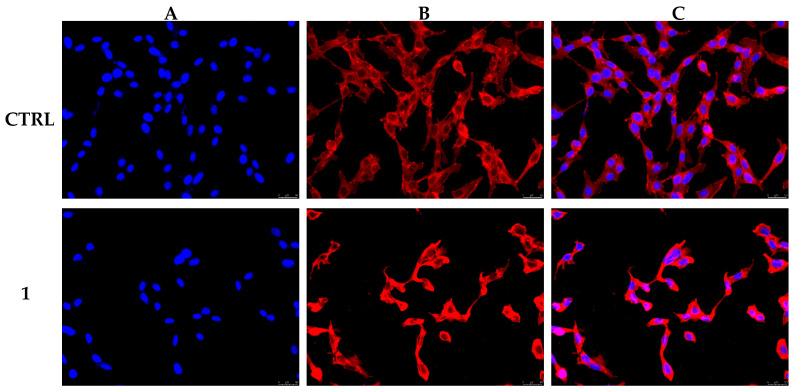
Actin immunofluorescence studies were conducted on MDA-MB-231 cells treated with **1** (0.3 µM) or vehicle (CTRL, DMSO) for 24 h. Cells were processed as detailed in the Section 2. Control cells (CTRL) showed a normal actin cytoskeleton organization, whereas under **1** exposure, the actin network appeared irregularly arranged and packed into the cell cytoplasm. Panels (**A**): nuclear DAPI staining (λex/λem = 350/460 nm); Panels (**B**): β-actin (Alexa Fluor^®^ 568; λex/λem = 644/665 nm); Panels (**C**): overlay. Images were taken at 20× and show representative fields.

Since the cell cytoskeleton is a dynamic complex made of different filamentous proteins within the cytoplasm, we also wondered whether the perturbation of the tubulin network, caused by compound **1** exposure, could also influence another major component, *viz.*, actin [48]. Indeed, the reorganization of the actin cytoskeleton was demonstrated to play a fundamental role in tumor cell invasion and metastasis formation. Moreover, the nuclear actin pool, whose presence was ascertained, is a component of chromatin remodeling complexes and is strictly related to gene expression [50]. Thus, we checked the status of the actin network in MDA-MB-231 cells by the means of immunostaining studies, under the same experimental conditions used for the tubulin investigations. Figure 8 shows the obtained outcomes, where it is possible to see that in the control experiment, MDA-MB-231 cells possess actin filaments that are thin and normally distributed within the cytoplasm (panel A, CTRL). On the contrary, compound **1** treatment induced the formation of thicker actin bundles that resulted in packing into the cytoplasm (panel B, **1**) and, consequently, a dramatic change in cell morphology. It is known that actin filaments and microtubules work together to ensure cell shape, transport, and mechanical forces, allowing the diverse functions. The concept that actin filaments and microtubules were separate entities, with different regulatory proteins and distinct cellular localization, was outworn by then [51]. Instead, different studies demonstrated a direct and coordinated relationship and that microtubules influence specific actin array formation or, vice versa, that F-actin can modulate the microtubule dynamics [52,53,54,55]. These studies strongly suggested that both the polymer systems are intrinsically intertwined. All considered, we believe that the demonstrated stabilization of microtubules, induced by compound **1** treatment in MDA-MB-231 cells, strongly influenced, as well, the intracellular actin network and pushed cancer cells to die, activating the apoptotic mechanism.

### 3.5. In Vivo Studies

**Figure 9:**
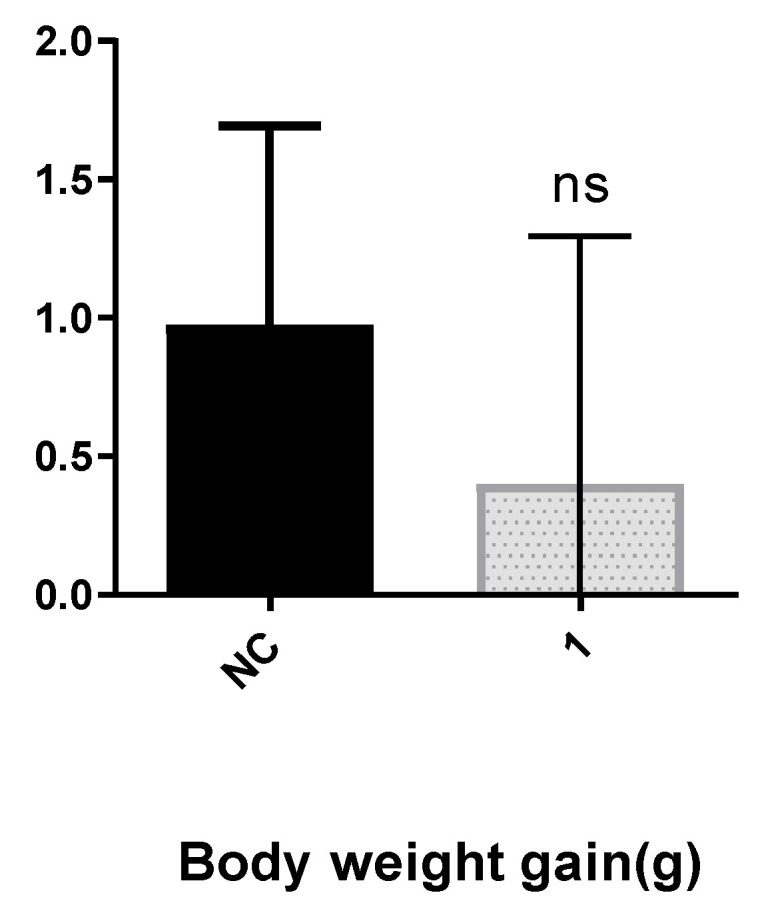
Body weight variation in mice after 1 week of treatment was ns (not significant), compared to the negative control by t-test; NC: negative control; **1**: compound **1**-treated group.

The in vivo evaluation of compound **1** was conducted through a one-week intraperitoneal administration at a dosage of 20 mg/kg body weight in Balb/c mice. Throughout the study period, there were no observable toxic effects related to treatment with compound **1**. The mice maintained normal behavioral patterns, indicative of good health, and their body weights remained stable. Statistical analysis showed no significant differences in body weight change when comparing the compound **1**-treated group to the negative control (NC) group (*p* > 0.05) (Figure 9).

**Figure 10:**
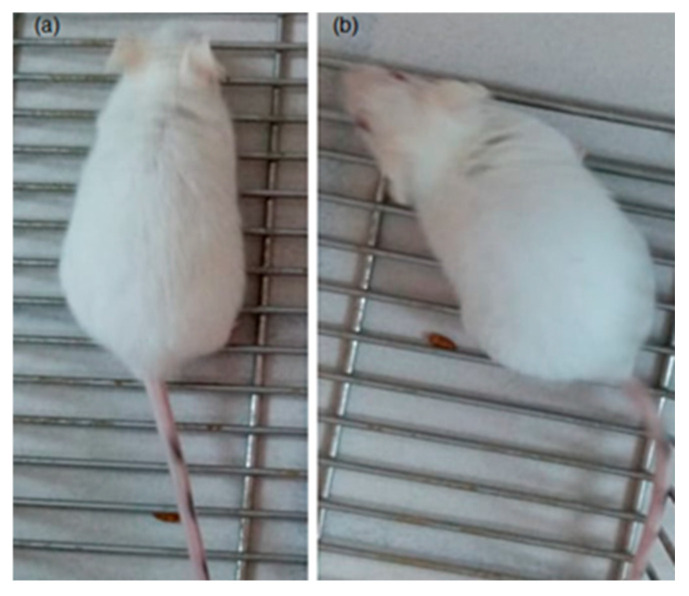
Representative photos of mice behaviors: (**a**) healthy and normal behaviors were registered for negative control (NC) and (**b**) the compound **1** group.

**Figure 11:**
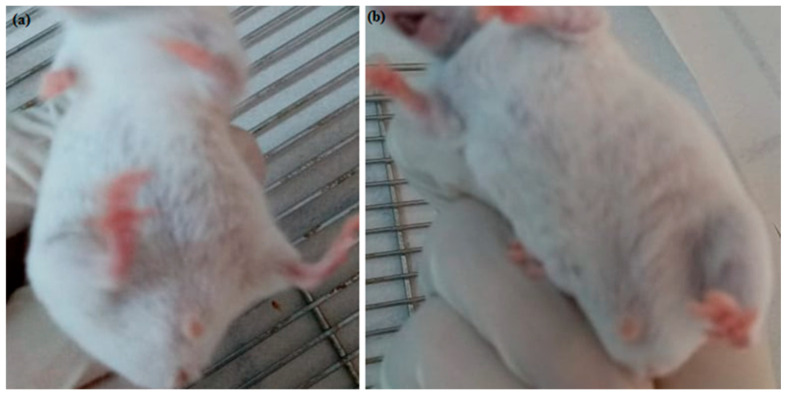
Representative photos for mice NC (**a**) and the compound **1**-treated group (**b**); healthy global appearance; no visible difference or abnormalities at the injection site.

Representative photographs (Figure 10 and Figure 11) further corroborated these findings, demonstrating an absence of visible abnormalities or signs of distress in the compound **1**-treated animals. This aligns with findings in earlier studies that reported minimal side effects of similar compounds on murine models [56,57].

**Figure 12:**
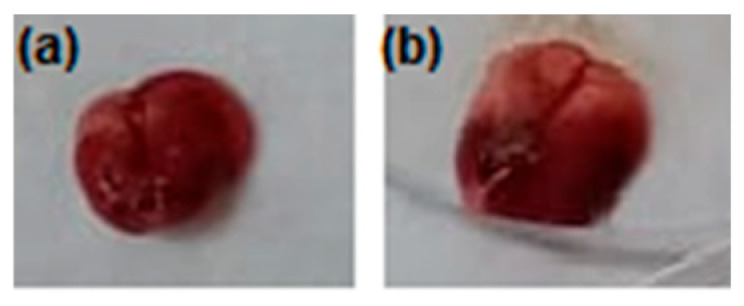
Effect of compound **1** on lung morphology: (**a**) negative control with normal lung morphology; (**b**) compound **1**-treated group showed normal morphologic features of lung, while visible weight gain for the organ was detected.

Post-treatment analysis of organ weights, including spleen, liver, brain, heart, kidney, and lung, revealed no significant differences between the compound **1**-treated group and the negative control group (*p* > 0.05). However, there was a notable increase in the lung coefficient for the compound **1**-treated group, which may suggest a potential physiological response to the treatment (Table 2 and Figure 12). Importantly, the examination of lungs from the compound **1**-treated group indicated normal evidence and no visible pathological changes (Figure 11). Similar findings have been documented in studies examining the safety profiles of various therapeutic agents in preclinical settings [58,59].

**Figure 13:**
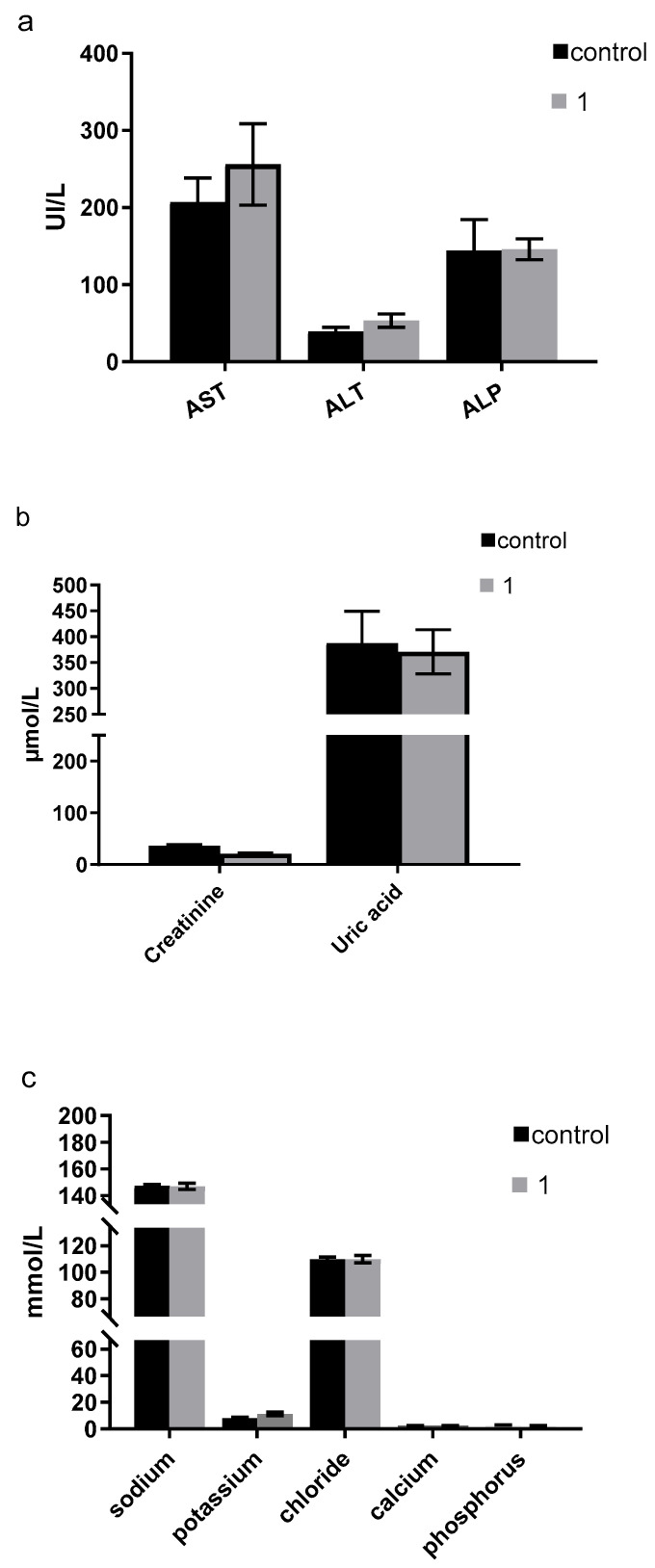
Biochemical analyses of the serum from groups of mice untreated or treated with compound **1;** (**a**) aspartate aminotransferase (AST), alanine aminotransferase (ALT), and alkaline phosphatase (ALP); (**b**) creatinine and uric acid; and (**c**) sodium, potassium, chloride, calcium, and phosphorus serum levels were measured in the two groups of mice (control and compound **1**-treated) and after 1 week.

Comprehensive biochemical analyses demonstrated that serum levels of transaminases (aspartate transaminase [AST] and alanine transaminase [ALT]), creatinine, uric acid, and the electrolyte profiles (sodium, potassium, and chloride) were comparable between the compound **1**-treated group and the control group (*p* > 0.05) (Figure 13). These results suggest the absence of hepatotoxicity and nephrotoxicity associated with **1**, which is consistent with findings from similar studies indicating favorable biochemical profiles for compounds targeting similar pathways [36,37].

**Figure 14:**
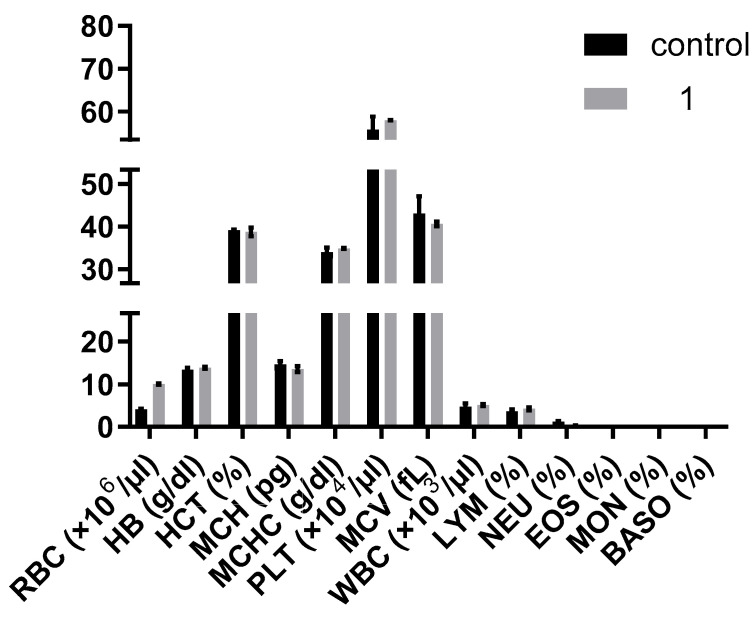
Hematological analysis of mice blood from groups of mice untreated and treated with compound **1;** RBC: red blood cells; HB: hemoglobin; HCT: hematocrit; MCH: mean corpuscular hemoglobin; MCHC: mean corpuscular hemoglobin concentration; PLT: platelet count; MCV: mean corpuscular volume; WBC: white blood cells; LYM: lymphocytes; NEU: neutrophils; EOS: eosinophils; MAC: macrophages; BASO: basophiles.

Hematological analyses also showed no significant differences in white blood cell counts, red blood cell parameters—such as RBC, hemoglobin (HB), hematocrit (HCT), mean corpuscular volume (MCV), mean corpuscular hemoglobin (MCH), and mean corpuscular hemoglobin concentration (MCHC)—or platelet counts between the compound **1**-treated and control groups (*p* > 0.05) (Figure 14). This indicates that **1** does not induce hematological toxicity even at the administered dosage and duration, corroborating the safety profiles observed in other pharmacologic studies [60,61].

The outcomes of these studies indicate that **1** exhibits a favorable safety profile in Balb/c mice following a one-week intraperitoneal administration at a dosage of 20 mg/kg body weight. The absence of observable toxic effects, stable body weight, and normal behavioral patterns during the treatment period suggest that **1** has a low potential for acute toxicity, aligning with findings from other studies on other compounds [62,63]. In the context of organ weight analysis, the lack of significant differences between the treated and control groups further supports the conclusion that **1** does not adversely impact vital organ functions within the assessed timeframe. The observed increase in lung coefficients may indicate a physiological response. Previous research has reported similar phenomena where certain therapeutic agents can lead to physiological adaptations in the lung due to changes in vascular dynamics or inflammation [64]. Biochemical parameter assessments revealed no significant changes in serum levels of transaminases (AST and ALT), creatinine, uric acid, or electrolyte profiles. This suggests the absence of hepatotoxicity or nephrotoxicity associated with **1**, which is consistent with other studies demonstrating the safety of novel therapeutic agents in preclinical models [65]. The favorable biochemical profile indicates that **1** might be a promising candidate for further therapeutic development. Furthermore, results from hematological analyses indicated that **1** does not induce hematotoxicity. The hematological parameters, including white blood cell counts and red blood cell indices, remained stable, reinforcing the compound’s potential for safety.

### 3.6. Compound ***1*** Stability

The hydrolytic chemical stability is an important step in early drug discovery for ensuring the potential development of compounds useful in therapy [66]. Thus, the hydrolytic stability of compound **1** was determined in DMSO-d6/D2O (90:10) solution at 37 °C by ^1^H NMR spectroscopy. The obtained spectra were reported in Figure S1 (Supplementary Material), where it is possible to see that no changes were recorded, even after 24 h, demonstrating that the compound is stable. Furthermore, it is stable in the solid state even years after synthesis.

## 4. Conclusions

Herein, a bis-carbazole derivative, *N*,*N*’-bis-(6-bromo-1,4-dimethyl-9*H*-carbazol-3-ylmethylene)-hydrazine (**1**), was studied, and its anticancer properties and the underlying mechanisms have been disclosed. Particularly, this compound exerted a good activity toward two breast cancer cell lines, being more active against the highly aggressive MDA-MB.231 cells without affecting the viability of the normal counterpart, MCF-10A cells. The DNA damage and the impairment of the cytoskeleton functions, interfering with the tubulin polymerization and actin arrangement, together with the activation of caspase-8 and -9, were found to be involved. These events, together with the activation of the pro-apoptotic Bid protein, led to the detected apoptotic cancer cell death. Noteworthily, **1** administration in Balb/c mice did not cause organ injury or alteration of the hematological parameters, suggesting a safe profile after intraperitoneal administration for a week at the adopted dosage. These features suggest a high potential and a favorable risk-benefit profile of this compound to be exploited in the treatment of cancer.

## Figures and Tables

**Table 1:** Compound **1**, Ellipticine, Vinblastine, and Paclitaxel IC_50_ values (µM) calculated for the breast cancer cells, MCF-7 and MDA-MB-231, and the normal one, MCF-10A. Values are expressed as the mean ± standard deviation of three different experiments, performed in triplicate. Selectivity Index (SI) values for both compounds have been reported, as well.

		IC_50_ (µM)		SI	
	MCF-7	MDA-MB-231	MCF-10A	MCF-7	MDA-MB-231
**Compound 1**	0.6 ± 0.3	0.3 ± 0.2	>100	>166.7	>333.3
**Ellipticine**	1.3 ± 0.2	1.9 ± 0.1	1.2 ± 0.3	0.9	0.8
**Vinblastine**	(36.8 ± 0.7) × 10^−3^	(130.2 ± 1.1) × 10^−3^	(17.2 ± 0.6) × 10^−3^	0.13	0.47
**Paclitaxel**	(1.3 ± 0.7) × 10^−2^	(1.9 ± 0.5) × 10^−2^	(6.4 ± 0.8) × 10^−1^	33.7	49.2

**Table 2:** Change in organ weight coefficient [10× g (organ)/g(animal)].

	Organ Coefficient (mg/10 g) = 10 × Body Weight/Weight of the Mouse					
	Spleen	Liver	Lung	Brain	Heart	Kidney
NC	0.056 ± 0.019	0.48 ± 0.047	0.08 ± 0.018	0.133 ± 0.025	0.057 ± 0.007	0.069 ± 0.006
1	0.07 ± 0.02	0.54 ± 0.03	0.11 ± 0.01	0.13 ± 0.03	0.06 ± 0.01	0.07 ± 0.01

## Data Availability

Data are contained within the article/Supplementary Material.

## Supplementary Materials

The following supporting information can be downloaded at https://www.mdpi.com/article/10.3390/pharmaceutics17040415/s1. Figure S1: The hydrolysis stability of compound **1** was determined in DMSO-d6/D2O (90:10) solution at 37 °C by 1H NMR spectroscopy

## Abbreviations

MTAs	microtubule-targeting agents
MTT	3-(4,5-dimethylthiazol-2-y1)-2,5-diphenyltetrazolium Bromide
DMSO	dimethyl sulfoxide
TUNEL	terminal deoxynucleotidyl transferase dUTP nick end labeling
DAPI	4′,6-diamidino-2-phenylindole
SD	standard deviations
RMSD	root mean square deviation
AST	aspartate transaminase
ALT	alanine transaminase
ALP	alkaline phosphatase
RBC	red blood cell
HB	hemoglobin
HCT	hematocrit
MCH	mean corpuscular hemoglobin
WBC	white blood cell
MCV	mean corpuscular volume
MCHC	mean corpuscular hemoglobin concentration
PLT	platelet
WBC	white blood cells
LYM	lymphocytes
NEU	neutrophils
EOS	eosinophils
MAC	macrophages
BASO	basophile
OD	optical density
NC	negative control
