# Supplementary material for: Anti-Breast Cancer Properties and In Vivo Safety Profile of a Bis-Carbazole Derivative
Source: Pharmaceutics. 2025 Mar 25;17(4):415. doi: 10.3390/pharmaceutics17040415 (PMC12030284; doi:10.3390/pharmaceutics17040415)
Supplement: Supplementary file 1 [file pharmaceutics-17-00415-s001.zip › pharmaceutics-3513736-supplementary.pdf]

Supplementary material

Anti-Breast Cancer Properties and In Vivo Safety Profile of a Bis-Carbazole Derivative

Jessica Ceramella [1], Camillo Rosano [2], Domenico Iacopetta [1,*], Iméne Ben Toumia [2,3,4], Leila Chekir-Ghedira [3], Mouna Maatouk [3], Annaluisa Mariconda [5], Pasquale Longo [6], Patrick Dallemagne [7], Christophe Rochais [7] and Maria Stefania Sinicropi [1]

1 Department of Pharmacy, Health and Nutritional Sciences, University of Calabria, Via Pietro Bucci,
87036 Arcavacata di Rende, Italy; jessica.ceramella@unical.it (J.C.); s.sinicropi@unical.it (M.S.S.)
2 U.O. Proteomica e Spettrometria di Massa, IRCCS Ospedale Policlinico San Martino,
Largo Rosanna Benzi, 10, 16132 Genova, Italy; camillo.rosano@hsanmartino.it (C.R.); ben.toumia.imene@gmail.com (I.B.T.)
3 Laboratory of Bioactive Natural Substances and Biotechnology, Faculty of Dentistry of Monastir, University of Monastir, Monastir 5000, Tunisia; chekir@yahoo.fr (L.C.-G.); maatoukmouna@yahoo.fr (M.M.)
4 Laboratory of Molecular and Cellular Biology, Faculty of Dental Medicine of Monastir, University of Monastir, Monastir 5000, Tunisia
5 Department of Basic and Applied Sciences, University of Basilicata, Via dell'Ateneo Lucano, 10, 85100 Potenza, Italy; annaluisa.mariconda@unibas.it
6 Department of Chemistry and Biology "A. Zambelli", University of Salerno, Via Giovanni Paolo II, 132, 84084 Fisciano, Italy; plongo@unisa.it
7 Université Caen Normandie, Normandie University, CERMN UR4258, F-14000 Caen, France; patrick.dallemagne@unicaen.fr (P.D.); christophe.rochais@unicaen.fr (C.R.)
* Correspondence: domenico.iacopetta@unical.it

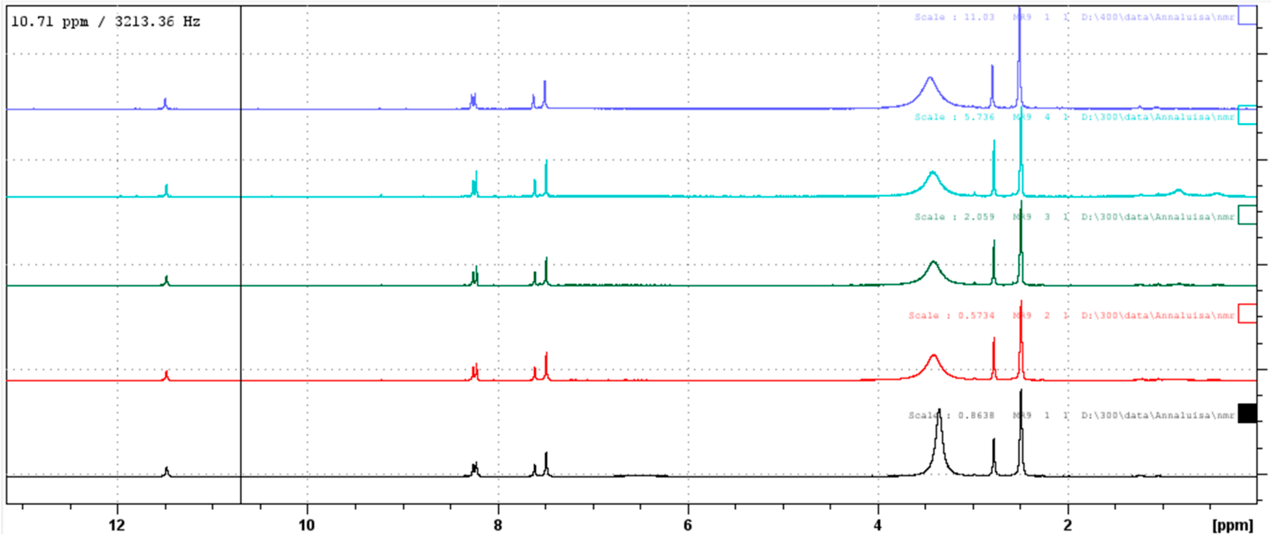

Figure S1. The hydrolysis stability of compound **1** was determined in DMSO-d6/D2O (90:10) solution at 37 °C by 1H NMR spectroscopy.
